# Global Studies of Using Fecal Biomarkers in Predicting Relapse in Inflammatory Bowel Disease

**DOI:** 10.3389/fmed.2020.580803

**Published:** 2020-12-17

**Authors:** Fang Liu, Seul A. Lee, Stephen M. Riordan, Li Zhang, Lixin Zhu

**Affiliations:** ^1^Department of General Surgery and Central Laboratory, The First Affiliated Hospital of Anhui Medical University, Hefei, China; ^2^School of Biotechnology and Biomolecular Sciences, University of New South Wales, Sydney, NSW, Australia; ^3^Gastrointestinal and Liver Unit, Prince of Wales Hospital, University of New South Wales, Sydney, NSW, Australia

**Keywords:** inflammatory bowel disease, Crohn's disease, ulcerative colitis, fecal biomarkers, prediction, calprotectin, lactoferrin, S100A12

## Abstract

Inflammatory bowel disease (IBD) is a chronic inflammatory condition of the gastrointestinal tract mainly comprising two forms including Crohn's disease (CD) and ulcerative colitis (UC). IBD is a lifelong relapsing remitting disease and relapses occur at random patterns which are unpredictable. Fecal biomarkers have been increasingly used to assess disease activity in IBD due to their positive correlations with intestinal inflammation. Recent studies have also assessed the use of fecal biomarkers in predicting relapse and post-operative recurrence. This review provides information from global studies of using fecal calprotectin, lactoferrin and S100A12 to predict relapse in IBD. Strategies for further studies and the use of these fecal biomarkers for personalized management in IBD are also discussed.

## Introduction

Inflammatory bowel disease (IBD) is a chronic inflammatory condition of the gastrointestinal tract comprising of two major subsets, Crohn's disease (CD) and ulcerative colitis (UC) (1). Inflammatory bowel disease is a lifelong disease and patients often experience multiple episodes of relapse and remission. Relapses in IBD occur at a random pattern, which are unpredictable. Endoscopy is not used routinely for disease monitoring due to its invasiveness and cost. Current monitoring of disease relapses in patients with IBD is symptom based (1). In order to improve patient management, various studies have assessed the use of fecal biomarkers in predicting disease relapse (2).

Fecal biomarkers have attracted a great attention owning to their non-invasiveness and cost effectiveness. Fecal biomarkers used in IBD are bioproducts resulted from inflammatory responses in the intestinal mucosa. Calprotectin is the most studied fecal biomarker. Lactoferrin, S100A12 and other fecal biomarkers have also been examined in recent years. Most of the studies have reported that these biomarkers correlate well with the endoscopic score and histological inflammation in patients with IBD (3–12).

Recent studies have also assessed the use of fecal biomarkers in predicting relapse and post-operative recurrence. In this review article, we provide comprehensive and updated information from global studies on the use of fecal calprotectin, lactoferrin and S100A12 to predict relapse in IBD. We have also discussed strategies for further studies and the use of these fecal biomarkers for personalized management in IBD.

## Biology of Calprotectin, Lactoferrin, and S100A12

Fecal biomarkers used in IBD are either actively secreted by or released from necrotic immune cells during inflammatory responses at the intestinal mucosa. They have a wide variety of biological functions including antimicrobial activity, proinflammatory activity, degradation of extracellular matrix and intracellular pathogens, as well as cellular and metabolic activities.

### Calprotectin

Calprotectin is a cytoplasmic protein prominently found in neutrophils that accounts for more than 40% of the cytosolic proteins in neutrophils, and to a lesser extent in monocytes and macrophages. Calprotectin is released to extracellular environment during inflammatory responses upon neutrophil activation or necrosis and induces neutrophil chemotaxis and adhesion. Calprotectin is stable for up to 1 year when stored at −20°C, and stable for 7 days when stored at 4°C and room temperature (13–15).

The physiologically active conformation of calprotectin is a heterodimer complex consisting of S100A8 and S100A9 and both proteins belong to the S100 family. The S100A8 and S100A9 subunits consist of 93 and 113 amino acids with molecular weight of 10.8 and 13.2 kDa, respectively (16, 17). Each subunit is able to bind two calcium ions. In addition to the calcium binding site, each heterodimer displays two transition metal binding sites at the interface of S100A8/S100A9, the first site binds manganese and zinc, while the second site binds zinc only (18–21).

As a metal chelating agent, calprotectin binds transition metals with high affinity and efficiently sequester them away from invading microbial pathogens, thereby starves invading pathogens, limiting their growth and resulting in a process called “nutritional immunity” (22–25). At the site of infection, calprotectin is not only abundantly released by neutrophils, but also epithelial cells and other immune cells, thereby playing a critical role in host defense against various bacterial species such as *Listeria monocytogenes, Salmonella* Typhimurium, *Borrelia burgdorferi, Helicobacter pylori, Staphylococcus aureus*, as well as fungal pathogens including *Candida albicans* (26–33). Interestingly, some bacterial pathogens harbor mechanisms allowing them to evade the harmful environment created by calprotectin. For examples, *H. pylori* is able to alter its outer membrane *via* lipid A modification, thus evading the antimicrobial activity of calprotectin. The growth of *S*. Typhimurium was actually elevated over competing commensal microbes in the presence of calprotectin due to the presence of ZnuABC zinc transporter, which enables the bacterium to acquire zinc under zinc-limiting conditions (34, 35).

### Lactoferrin

Lactoferrin is present in most exocrine secretions such as milk, saliva, tears, mucosal secretions, and plasma (36). Secretory epithelia and neutrophils are the main sources of lactoferrin. Lactoferrin is stable for up to 7 days when stored at 4°C or room temperature (37–39).

Human lactoferrin is an 80 kDa glycoprotein containing ~700 amino acids. The single polypeptide chain forms two homologous globular domains, namely N-terminal lobe and C-terminal lobe, respectively, depending on their localization, and each terminal lobe contains two domains (N1, N2, C1, and C2), resulting in a deep cleft conformation for iron-binding (40).

Lactoferrin has antimicrobial activity. Lactoferrin binds free iron, which inhibits the growth of iron-dependent bacterial species and reduces bacterial biofilm formation (41). Lactoferrin can also bind to receptors on bacterial surface, which induces death of Gram-negative bacteria due to a disruption in the cell wall and inhibits the formation of bacterial biofilms. Under inflammatory conditions, the levels of lactoferrin are increased.

### S100A12

S100A12 is also a protein of the S100 family that is predominately expressed and secreted by neutrophils. Human S100A12 contains 91 amino acids with a molecular weight of 10.4 kDa and the protein is stable for 7–10 days when stored at room temperature (42–44). Similar to calprotectin, S100A12 is able to bind calcium, iron and zinc. As a metal chelating agent, S100A12 also has antimicrobial activity (45–47). Furthermore, S100A12 has chemotactic characteristic that recruits mast cells and monocytes to the site of inflammation (48–50). S100A12 is able to bind a number of cellular receptors. Recent evidence suggest that S100A12 stimulate proinflammatory responses in monocytes *via* Toll-like receptor 4, leading to upregulated monocyte expression of proinflammatory cytokines including interleukin (IL)-1β, IL-6, and IL-8 (51). S100A12 is overexpressed in inflammatory conditions.

## Calprotectin, Lactoferrin, and S100A12 In Predicting Relapse In IBD

The gold standard of defining clinical remission or relapse relies on endoscopic mucosal healing and histological scoring of inflammation. Majority of the quiescent IBD patients have residual inflammation in the colonic mucosa, and when the degree of inflammation reaches a critical level, symptomatic relapse occurs (52). Various research groups have examined the use of fecal biomarkers as predictive markers for relapse and they are summarized in Table 1. Most of these studies assessed calprotectin and few examined lactoferrin and S100A12. Of the 31 studies listed in Table 1, 29 studies examined calprotectin, three studies examined lactoferrin and one study examined S100A12. Some of these studies have examined multiple fecal biomarkers.

**Table 1:** Summary of studies investigating fecal biomarkers for the prediction of relapses in inflammatory bowel disease.

**References**	**Location**	Age median or mean^*^ (range)	**Disease**	**N**	**Time interval**	**Optimal cut-off**	Median/mean^*^		***P*-value**	**Sensitivity/** **specificity %**	**PPV/NPV %**	**Method**
							**Relapse**	**Non-relapse**				
**Calprotectin**												
Buisson et al. (53)	US	25.9^*^	CD	112	1 yr	100 μg/g	-	-	-	76/86	77/85	ELISA (Genova diagnostics)
			UC	48								
Ferreiro-Iglesias et al. (54)	Spain	44 (18–78)	CD	71	4 mons	>300 μg/g	477 μg/g	65 μg/g	<0.005	100/80	78.3/100	Lateral flow assay (Buhlmann)
			UC	24								
Kittanakom et al. (55)	Canada	CD: 14.6 *(11–17)*UC: 14.1 *(11–17)*	IBD	40	-	400 μg/g	-	-	-	100/75.9	58.8/100	ELISA (PhiCal)
					-	800 μg/g	-	-	-	100/72.4	55.6/100	Fluorescence enzyme immunoassay (Phadia)
					-	500 μg/g	-	-	-	100/72.4	55.6/100	ELISA (Buhlmann)
Diederen et al. (56)	Netherlands	*14.9 (all <18)*	IBD	114	6 mons	350 μg/g	370 μg/g	122 μg/g	0.003	82/79	41/96	-
Roblin et al. (57)	France	35	CD	119	6 mons	>250 μg/g and TLI <2 μg/mL	-	-	-	94/84	73/97	Lateral flow assay (Buhlmann)
Theede et al. (58)	Denmark	39^*^	UC	70	6 and 12 mons	321 mg/kg	-	-	-	46.7/85.5	46.7/85.5	ELISA (Buhlmann)
Ferreiro-Iglesias et al. (59)	Spain	46 (18–68)	IBD	53	2 mons	160 μg/g	332 μg/g^*^	110 μg/g^*^	<0.005	91.7/82.9	68.7/96.1	Lateral flow assay (Buhlmann)
		41 (18–43)	CD	33		160 μg/g	287 μg/g^*^	94 μg/g^*^	<0.005	87.5/84.0	66.9/94.8	
		51 (19–68)	UC	20		198 μg/g	420 μg/g^*^	136 μg/g^*^	<0.005	100/81.3	48.5/100	
Ferreiro-Iglesias et al. (60)	Spain	38 (24–64)	CD	30	4 mons	204 μg/g	625 μg/g	45 μg/g	<0.005	100/85.7	74.1/100	Lateral flow assay (Buhlmann)
Delefortrie et al. (61)	Belgium	43	CD	29	6 mons	183.5 μg/g	667 μg/g	109 μg/g	<0.05	100/76.2	61/100	Lateral flow assay (Buhlmann)
						124.5 μg/g	339.5 μg/g	71.4 μg/g	<0.05	87.5/66.66	50/93.5	Chemiluminescent immunoassay (Liaison). Samples extracted with Liaison extraction device
						106.5 μg/g	261.5 μg/g	37.6 μg/g	<0.05	87.5/95.2	87.5/95	Chemiluminescent immunoassay (Liaison). Samples extracted with weighing protocol
Mooiweer et al. (62)	Netherlands	50 (19–71)	CD	20	12 mons	56 μg/g^&^	284 μg/g	37 μg/g	<0.01	64/100	20/100	ELISA (Ridascreen)
			UC/IBD-U	52								
Yamamoto et al. (63)	Japan	35 (18–74)	UC	80	40 wks	Elevated level ≥55 μg/g	76.5 μg/g	15.5 μg/g	<0.0001	88/80	66/94	ELISA (Cell sciences)
Scaioli et al. (64)	Italy	40 (16–89)	UC	74	1 yr	193 μg/g	218 μg/g	48 μg/g	<0.01	65/98	92/88	ELISA (Calprest)
Yamamoto et al. (65)	Japan	35.1^*^ (20–75)	UC	80	12 mons	170 μg/g	173.7 μg/g^*^	135.5 μg/g^*^	0.02	76/76	-/-	ELISA (Cell sciences)
Jauregui-Amezaga et al. (66)	Spain	46^*^	UC	64	1 yr	250 μg/g	200 μg/g	75 μg/g	0.75	41/85	-/80	ELISA (Cerba internacional)
Naismith et al. (67)	UK	47^*^ (>18)	CD	92	12 mons	240 μg/g	414 μg/g	96 μg/g	0.005	80.8/74.4	28/97	ELISA (Buhlmann)
Vos et al. (68)	Belgium and Norway	48^*^ (19–79)	UC	87	52 wks	300 μg/g	125 μg/g^*^	27 μg/g^*^	<0.001	58.3/93.3	-/-	ELISA (PhiCal)
						Two consecutive measurements of >300 μg/g within 1 mon				61.5/100	-/-	
Lasson et al. (69)	Sweden	33 (18–74)	UC	69	1 yr	169 μg/g	263 μg/g	102 μg/g	0.009	64.4/70.8	80.6/51.5	ELISA (Buhlmann)
				67	2 yrs	262 μg/g	263 μg/g	124 μg/g	<0.05	51.1/81.8	85.2/45.0	
				67	3 yrs	262 μg/g	280 μg/g	118 μg/g	0.01	52.2/85.7	88.9/45.0	
Meuwis et al. (70)	France and Belgium	32	CD	79	28 mons	250 μg/g	-	-	-	-/-	-/-	ELISA (PhiCal)
van Rheenen et al. (71)	Netherlands	14.1^*^ (<18)	CD	31	3 mons	500 μg/g	-	-	-	67/81	-/-	ELISA (Calpro)
		13^*^ (<18)	UC	31								
Louis et al. (72)	France and Belgium	32 (>17)	CD	115	1 yr	300 μg/g	-	-	-	-/-	-/-	ELISA (PhiCal)
Laharie et al. (73)	France	30.4 (15–69)	CD	65	14 wks	130 μg/g	200 μg/g	150 μg/g	Ns	61/48	-/-	ELISA (Buhlmann)
						250 μg/g				43/57	-/-	
García-Sánchez et al. (74)	Spain	36.9^*^	CD	66	1 yr	200 μg/g	524 μg/g	123 μg/g	<0.01	80/65	46/88	ELISA (Calprest)
		40.4^*^	UC	69		120 μg/g	298 μg/g	105 μg/g	<0.01	81/63	49/88	
Kallel et al. (75)	Tunisia	33 (15–66)	CD	53	12 mons	340 μg/g	380.5 μg/g	155 μg/g	<0.001	80/90.7	-/-	ELISA (Calprest)
Sipponen et al. (76)	Finland	*12.9 (2–17)*	IBD	72	12 mons	108.5 μg/g	409 μg/g	282 μg/g	0.44	38/72	-/-	ELISA (PhiCal)
						100 μg/g				-/-	39.6/75	
Gisbert et al. (77)	Spain	43^*^	IBD	163	12 mons	150 μg/g	239 μg/g	136 μg/g	<0.001	69/69	30/92	ELISA (PhiCal)
			CD	89		150 μg/g	266 μg/g	145 μg/g	0.002	28/93	-/-	
			UC	74		150 μg/g	213 μg/g	126 μg/g	0.03	31/91	-/-	
D'incà et al. (78)	Italy	-	IBD	162	1 yr	130 μg/g	-	-	-	68/67	52/79	ELISA (Calprest)
		43 (18–77)	CD	65		130 μg/g	207 μg/g	88 μg/g	0.055	65/62	44/80	
		46 (15–80)	UC	97		130 μg/g	190 μg/g	49 μg/g	0.02	70/70	60/79	
Diamanti et al. (79)	Italy	-	IBD	73	3 yrs	275 μg/g	-	-	-	97/85	85/97	ELISA (Calprest)
		16 (1.5–18)	CD	32		462 μg/g	-	-	-	100/71	78/100	
		12 (6–18)	UC	41		275 μg/g	-	-	-	94/95	94/95	
Costa et al. (80)	Italy	35.7^*^	CD	38	12 mons	150 μg/g	220.1 μg/g	220.5 μg/g	0.395	87/43	50/83	ELISA (Calprest)
		41.2^*^	UC	41		150 μg/g	220.6 μg/g	67 μg/g	<0.0001	89/82	81/90	
Tibble et al. (81)	UK	33	CD	43	12 mons	100 μg/g	244 μg/g	84 μg/g	<0.0001	90/83	-/-	ELISA (In-house)
		49	UC	37			246 μg/g	58 μg/g	<0.0001			
**Lactoferrin**												
Yamamoto et al. (65)	Japan	35.1^*^ (20–75)	UC	80	12 mons	140 μg/g	161.5 μg/g^*^	130.7 μg/g^*^	0.03	67/68	-/-	Colloidal gold agglutination assay (Alfresa Pharma Corp.)
Gisbert et al. (77)	Spain	43^*^	IBD	163	12 mons	-	62%^∧^	35%^∧^	<0.05	62/65	25/90	ELISA (TechLab)
Walker et al. (82)	US	13.4^*^ (2–21)	IBD	55	2 mons	-	845 μg/g^*^	190 μg/g^*^	0.003	-/-	-/-	ELISA (TechLab)
**S100A12**												
Däbritz et al. (83)	Germany	37.4 (3.5–74.6)	IBD	181	Predicting relapse 8–12 wks earlier	0.43 μg/g	-	-	-	70/83	-/-	ELISA (In-house)
			CD	61								
			UC	120								

Time interval: cut-off values for predicting relapse within a specified period. Concentrations of fecal markers in relapsers and non-relapsers are expressed as mean

(*) or median. Age of patients are presented as mean (*) or median. Studies on pediatric patients are in italic.

& *Cut-off value for prediction of absence of relapse*.

∧ *Positive lactoferrin test was more frequent in relapsing than in non-relapsing patients. TLI, trough level of infliximab; IBD, inflammatory bowel disease; CD, Crohn's disease; UC, ulcerative colitis; IBD-U, inflammatory bowel disease-unclassified; PPV, positive predictive value; NPV, negative predictive value; Ns, Not statistically significant; Wk, week. Mon, month; Yr, year. -, information not available*.

The reported sensitivities, specificities and the cut-off values in different studies assessing fecal calprotectin as a biomarker in predicting relapse varied greatly. Of the 29 studies of calprotectin listed in Table 1, the sensitivities for predicting CD, UC, and IBD ranged from 28 to 100%, 31 to 100%, and 38 to 100%, respectively. The specificities for predicting CD, UC, and IBD ranged from 43 to 52%, 63 to 100%, and 69 to 100%, respectively. The cut-off values for CD, UC, and IBD varied from 106.5 to 462 μg/g, 120 to 321 μg/g, and 100 to 800 μg/g, respectively (Table 1). Twenty-one studies compared the levels of calprotectin of relapsed and non-relapsed patients, of which 18 studies (85.7%) found that the levels of fecal calprotectin in relapsed patients were significantly higher, indicating that the levels of fecal calprotectin reflect the levels of inflammation in the intestinal mucosal tissues. A meta-analysis by Mao et al. analyzed combined data from six studies in Table 1, comprising a total of 672 adult IBD patients (318 UC and 354 CD) (84). They reported that the pooled sensitivity and specificity of fecal calprotectin in predicting relapse in quiescent IBD to be 78 and 73%, respectively (84). However, this meta-analysis did not state the cut-off values of the pooled data, the cut-off values in the six original studies varied from 100 to 340 μg (74, 75, 77, 78, 80, 81).

The time intervals observed in studies examining fecal calprotectin in Table 1 were from 2 months to 3 years. More than 50% of these studies observed patients for a time interval of 1 year or above. The remaining studies observed patients for shorter terms such as 2, 4, or 6 months. There were no specific traits associated with observation term intervals in respect of cut-off values, sensitivities and specificities.

Most of the studies on fecal calprotectin in predicting IBD relapse were from Europe. Of the 29 studies examining calprotectin in Table 1, 23 were from Europe, two from North America, two from UK, one from Africa, and there were only two studies from Asian populations, both of which were from the same research group in Japan (63, 65).

Enzyme-linked immunosorbent assay (ELISA) was used in quantifying the levels of calprotectin in stools in 23 out of the 29 studies in Table 1. The remaining studies used other methods such as Lateral Flow Assay, chemiluminescent immunoassay, colloidal gold agglutination assay, and fluorescence enzyme immunoassay. The ELISA kits used by these studies were from eight different manufacturers and one study used in-house ELISA. The studies by Kittanakom et al. and Delefortrie et al. have compared different methods in quantifying fecal calprotectin for predicting relapse of IBD and CD, respectively (55, 61). Kittanakom et al. (55) reported the cut-off values of 400 and 500 μg/g when using ELISA kits supplied by two different manufacturers, however the cut-off was of a much higher value (800 μg/g) when fluorescence enzyme immunoassay was used. Delefortrie et al. showed cut-off values of 124.5 and 106.5 μg/g when the same chemiluminescent immunoassay was performed with different sample extraction methods, but the cut-off was much higher (183.5 μg/g) when Lateral Flow Assay was used (61). These results showed that variations can be introduced due to different detection methods used in various studies.

To date, only three studies have investigated the use of fecal lactoferrin in predicting relapse in IBD, of which only the study from Japan was able to identify an optimal cut-off value (65). However, this study did not find a statistically significant difference of fecal lactoferrin levels between relapsed and non-relapsed patients. The remaining two studies from Spain and US, although have found a significant difference of fecal lactoferrin levels between relapsed and non-relapsed patients, but they did not report optimal cut-off values for prediction of relapse (77, 82). Only one study had examined the use of S100A12 for predicting relapse in IBD. By using an in-house ELISA, Däbritz et al. showed that a cut-off value of 0.43 μg/g was able to predict relapse 8–12 weeks earlier with sensitivity and specificity being 70 and 83% respectively.

## Calprotectin, Lactoferrin, and S100A12 In Predicting Post-Operative Recurrence In CD

A non-invasive biomarker with predictive potential to identify patients without recurrence would be desirable to avoid post-operative endoscopies. In recent years, the use of fecal calprotectin in predicting post-operative recurrence in CD has been evaluated by various studies. Limited studies have also examined lactoferrin and S100A12. These studies are listed in Table 2.

**Table 2:** Summary of studies investigating fecal biomarkers for the prediction of post-operative recurrence in patients with Crohn's disease.

**References**	**Location**	Age median or mean^*^ (range)	**N**	**Time interval**	**Optimal cut-off**	Median/mean^*^		***P*-value**	**Sensitivity/** **specificity %**	**PPV/NPV %**	**Method**
						**With POR**	**Without POR**				
**Calprotectin**												
Cerrillo et al. (85)	Spain	40.7^*^ (18–74)	61	24 mons	160 μg/g	-	-	-	85/70	26/98	ELISA (Calprest)
Baillet et al. (86)	France	34.9^*^	30	1 yr	100 μg/g	354.8 μg/g^*^	114 μg/g^*^	0.0075	67/93	89/77	Lateral Flow Assay (Buhlmann)
Verdejo et al. (87)	Spain	46.2	86	<1 mon	62 μg/g	172.5 μg/g	75 μg/g	0.003	85.7/45.9	67.7/70.8	Lateral flow assay (Buhlmann)
Garcia-Planella et al. (88)	Spain	40	119	~24 mons	100 μg/g and 5 mg/L of CRP	205 μg/g^*^	94 μg/g^*^	<0.0001	82/53	54/81	ELISA (Calprest)
Wright et al. (89)	Australia and New Zealand	36	135	18 mons	135 μg/g	275 μg/g	72 μg/g	<0.001	87/66	56/91	ELISA (Buhlmann)
Lopes et al. (90)	Portugal	45^*^	99	25 mons^#^	100 μg/g	196.5 μg/g	42.1 μg/g	<0.001	74/75	61/91	Fluorescence enzyme immunoassay (Thermo Fisher Scientifi)
Hukkinen et al. (91)	Finland	*13.6 (≤18)*	22	5.7 yrs^#^	139 μg/g	-	-	-	73/64	68/70	ELISA (PhiCal)
					Increase of 79 μg/g	-	-	-	73/71	73/71	
Herranz Bachiller et al. (92)	Spain	48.6^*^	97	-	60 μg/g	192.45 μg/g	94.39 μg/g	0.0001	88/58	51.73/83.9	ELISA (Calprest)
Yamamoto et al. (93)	Japan	32 (21–48)	30	24 mons	140 μg/g	199 μg/g	82.5 μg/g	0.002	75/91	75/91	Colloidal gold agglutination assay (Alfresa Pharma Corp.)
Boschetti et al. (94)	France	39.3^*^ (18–70)	86	18 mons	100 μg/g	473 μg/g^*^	115 μg/g^*^	<0.0001	95/54	69/93	ELISA (Buhlmann)
Lasson et al. (95)	Sweden	36 (17–63)	30	1 yr	100 μg/g	227 μg/g	189 μg/g	0.25	85/35	50/75	ELISA (Buhlmann)
					200 μg/g				54/53	47/60	
					250 μg/g				46/53	43/56	
^∧^Yamamoto et al. (96)	Japan	32^*^	20	12 mons	140 μg/g	229.5 μg/g^*^	102.3 μg/g^*^	0.005	70/70	70/70	ELISA (Cell sciences)
Lobatón et al. (97)	Spain	40	115	-	272 μg/g	788.5 μg/g^*^	100 μg/g^*^	<0.001	79/97	98/76	Lateral flow assay (Buhlmann)
					274 μg/g	1211.9 μg/g^*^	101.8 μg/g^*^	<0.001	77/97	98/75	ELISA (Buhlmann)
Yamamoto et al. (98)	Japan	-	20	12 mons	170 μg/g	-	-	-	83/93	-/-	ELISA (Manufacturer not specified)
Orlando et al. (99)	Italy	38	50	3 mons	200 mg/L	-	-	-	63/75	70/68	ELISA (Calprest)
**Lactoferrin**												
Wright et al. (89)	Australia and New Zealand	36	135	18 mons	3.4 μg/g	5.7 μg/g	1.6 μg/g	0.007	70/68	53/81	ELISA (TechLab)
Lopes et al. (90)	Portugal	45^*^	99	25 mons^#^	7.25 μg/g	23.27 μg/g	2 μg/g	<0.001	74/68	61/91	ELISA (TechLab)
^∧^Yamamoto et al. (96)	Japan	32^*^	20	12 mons	125 μg/g	161.4 μg/g^*^	83.7 μg/g^*^	0.02	70/60	64/67	Colloidal gold agglutination assay (Alfresa Pharma Corp.)
Yamamoto et al. (98)	Japan	-	20	12 mons	140 μg/g	-	-	-	67/71	-/-	Colloidal gold agglutination assay (Manufacturer not specified)
**S100A12**												
Wright et al. (89)	Australia and New Zealand	36	135	18 mons	10.5 μg/g	2.0 μg/g	0.8 μg/g	0.188	91/12	35/71	ELISA (In-house)

Majority of the studies have examined the use of fecal biomarkers for prediction of endoscopic recurrence, except the study performed by Yamamoto et al. (96)

(∧) which was on clinical recurrence. Time-interval: median

(#) or maximum follow up period. Concentrations of fecal markers in patients with and without POR are expressed as mean

(*) or median. Age of patients are presented as mean (*) or median. Studies on pediatric patients are in italic. IBD, inflammatory bowel disease; CD, Crohn's disease; UC, ulcerative colitis; POR, post-operative recurrence; CRP, C-reactive protein; PPV, positive predictive value; NPV, negative predictive value; -, information not available.

These studies again reported varied sensitivities, specificities and cut-off values. Studies examining calprotectin reported sensitivities between 46 and 95% and specificities between 45.9 and 97%. The cut-off values also ranged from 60 to 274 μg/g. In the study by Lasson et al. (95) three different cut-off values (100, 200, and 250 μg/g) were assessed, and the corresponding sensitivities were 85, 54, and 46%, respectively. Nevertheless, this study did not detect a significantly different levels of fecal calprotectin in patients with and without post-operative recurrence while the other studies did (Table 2). A meta-analysis performed by Tham et al. on examining the use of fecal calprotectin for detection of post-operative endoscopic recurrence in CD showed that a significant threshold effect was observed for fecal calprotectin values of 50, 100, 150, and 200 μg/g; while the optimal diagnostic accuracy was obtained for fecal calprotectin value of 150 μg/g, with a pooled sensitivity and specificity being 70 and 69%, respectively (100).

Four studies have examined lactoferrin, which all showed significantly different fecal lactoferrin levels in patients with and without post-operative recurrence. However, the cut-off values ranged from 3.4 to 140 μg/g (Table 2). Only one study has examined S100A12 in pediatric patients using an in-house ELISA, which reported a sensitivity of 90% and specificity of 12%, and no significant difference in fecal S100A12 levels was observed in patients with and without post-operative recurrence (Table 2).

## Discussion and Suggestions

Studies from diverse geographical regions of the world, mainly from Europe, have examined the use of fecal biomarkers in predicting disease relapse and post-operative recurrence in patients with IBD. Calprotectin is the most studied marker, and several studies also examined lactoferrin and few have investigated S100A12. The consistent information from these studies is that the level of calprotectin increases along with the intestinal mucosal inflammation, which is consistent with the biological functions of this protein. However, whether it can be used to predict disease relapse and post-operative recurrence is inconclusive from the current studies.

Several factors from these studies have contributed to the uncertainty of using fecal biomarkers in predicting disease relapse and post-operative recurrence. Firstly, the cut-off values used in these studies varied remarkably, making it difficult to draw reliable conclusion. Secondly, different detection methods were used, which may produce inconsistent results. Thirdly, the time intervals observed in different studies were random, which again makes it difficult to compare the results between studies. Further studies therefore are warranted to determine whether these fecal biomarkers are reliable predicative markers in the management of IBD. We suggest the following strategies.

### Use Fecal Biomarkers as Markers for Personalized Management in IBD

The degree of mucosal inflammation, the level of inflammation that can cause clinical symptoms and the response to different therapeutic agents in individual patients with IBD vary greatly. Given this, fecal biomarkers are perhaps best used in personalized management. Fecal samples can be collected at different stages of IBD in individual patients and the levels of fecal biomarkers can then be measured. Changes in levels of fecal biomarkers can be used to monitor and predict disease progress in individual patients, which may lead to an enhanced patient management.

### Coordinated Multi-Center Analysis

Coordinated multi-center studies from different geographic regions are needed in order to determine whether fecal biomarkers can be used as reliable predictive markers for patients with IBD globally. Samples in different centers should be collected at multiple but consistently defined timepoints. Given that ELISA was the most commonly used quantification method in previous studies, perhaps this method should still be used. However, ELISA kits provided by different manufacturers should be compared. Consistently defined cut-off values should be used for data analysis. This approach is more likely to produce conclusive data regarding whether fecal biomarkers can be used as cohort markers to predict disease relapse in patients with IBD.

## Author Contributions

FL played a major role in writing the manuscript. LZhu and LZha conceived the project. LZhu, LZha, SL, and SR provided critical feedback and helped in editing the manuscript. All authors have approved the final version of the manuscript.

## Conflict of Interest

The authors declare that the research was conducted in the absence of any commercial or financial relationships that could be construed as a potential conflict of interest.
